# A succinct technique for the extraction of the proximal femoral nail anti-rotation (PFNA) after unlocking failure: a case report

**DOI:** 10.1186/s13018-020-01821-4

**Published:** 2020-08-06

**Authors:** Ming-de Cao, Zhong-Meng Yang, Hua-Ding Lu

**Affiliations:** Department of Orthopedics, The Fifth Affiliated Hospital of Sun Yat-Sen University, Zhuhai, 519000 Guangdong China

**Keywords:** Proximal femoral nail anti-rotation, Implant removal, Unlocking failure

## Abstract

**Introduction:**

Proximal femoral nail anti-rotation (PFNA) is a routine method to deal with intertrochanteric fractures in the elder population. It is challenging to remove PFNA in some cases as a result of stripping of blade heads. In this case presentation, we describe a novel technique using commonly available instruments that can be used to remove stripped, even broken anti-rotation blade where conventional methods have failed.

**Methods:**

The subject underwent a PFNA removal surgery 15 months after the previous fixation. We encountered difficulties using the regular instrument to remove the anti-rotation blade. A 5-mm tungsten carbide bur was used to drill a single cortical hole at the end of the blade. Then double-strand steel wire was threaded through the hole, and the distal part was shaped into a circle which could tie to the extraction screw. Slide Hammer was applied to gently knock out the blade along the anatomical direction of the femoral neck.

**Results:**

The technique helped us successfully remove the anti-rotation blade and provided the patient with a satisfactory result.

**Conclusion:**

The use of a tungsten reamer and steel wire loop to remove the proximal femoral anti-rotation blade may provide a cost-effective and straightforward method of dealing with extraction failure.

## Introduction

Owing to an aging population, the incidence of proximal femoral fractures continues to rise. Intertrochanteric fractures are of the most common fracture types, which are routinely treated with proximal femoral nail anti-rotation (PFNA) today. Compared with plate-screw fixation, various researches suggest that PFNA is a better choice for the treatment for unstable peritrochanteric fractures [1]. With the enhancement of the technique, the effects have increased, and complications have reduced. However, when we intend to remove the implant, orthopedic surgeons are frequently faced with the challenge of removing the anti-rotation blade when the blind nut of it stripped. None of simple and effective methods for extraction of PFNA has been previously described. We describe a novel technique using a commonly available instrument that can be used to remove stripped even broken anti-rotation blade where regular methods have failed.

## Surgical technique and case presentation

**Fig. 1:**
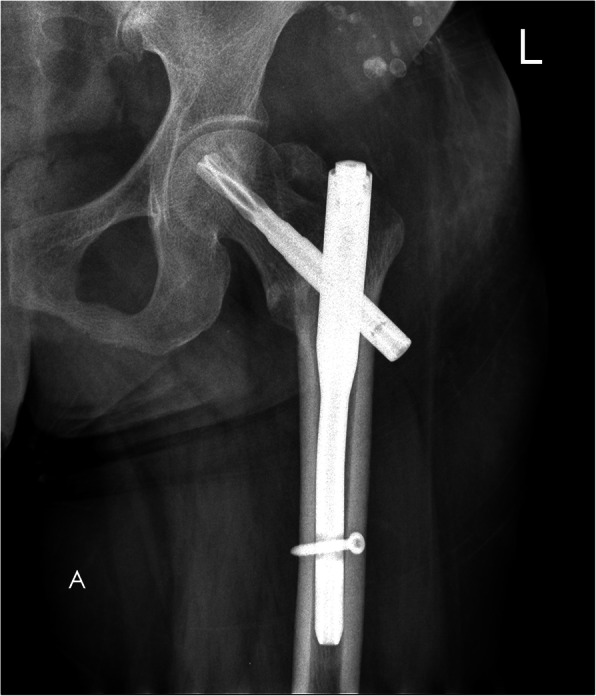
The anteroposterior radiograph of left hip after the PFNA fixation

**Fig. 2:**
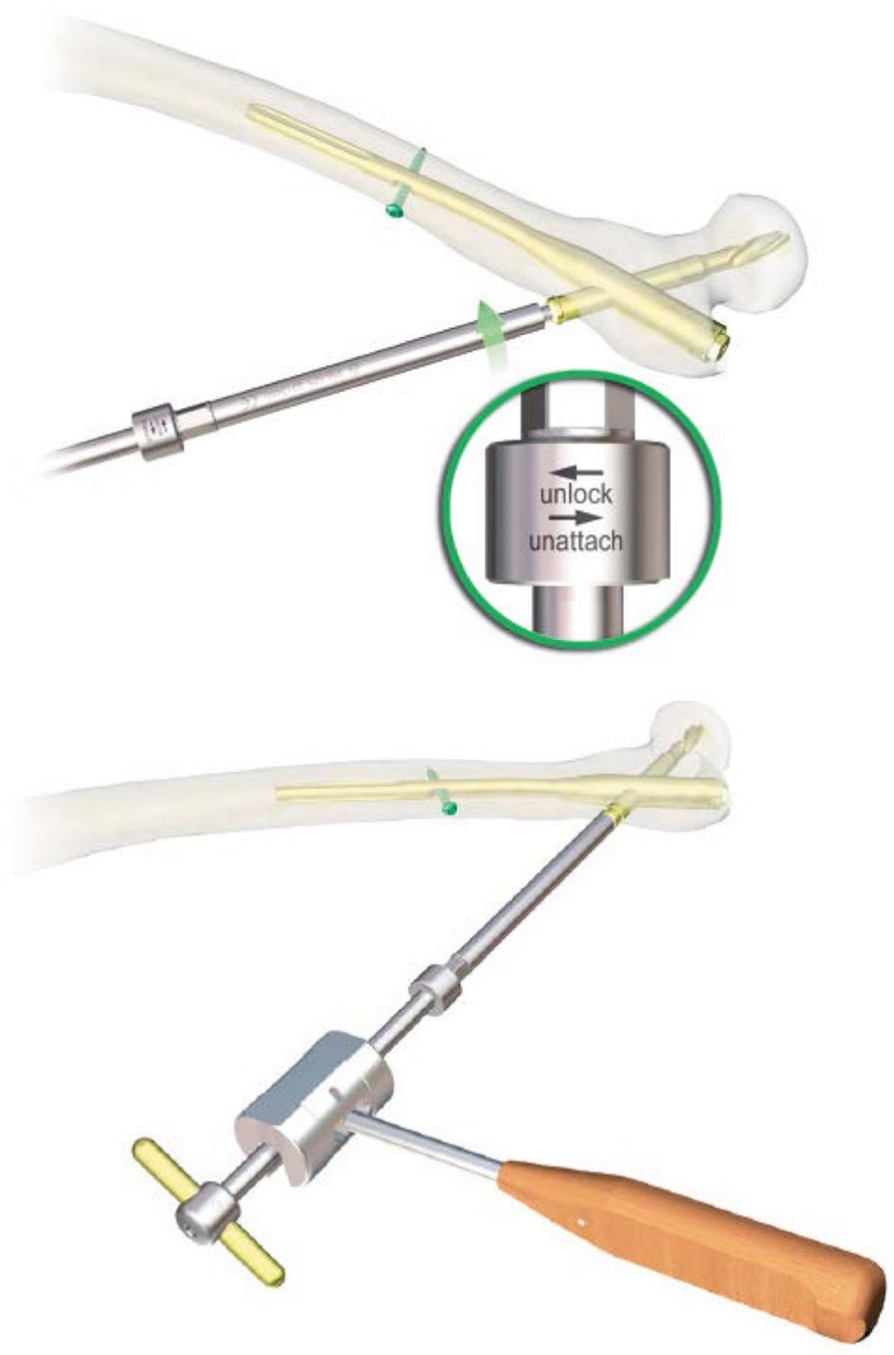
The standard procedure of PFNA blade removal

**Fig. 3:**
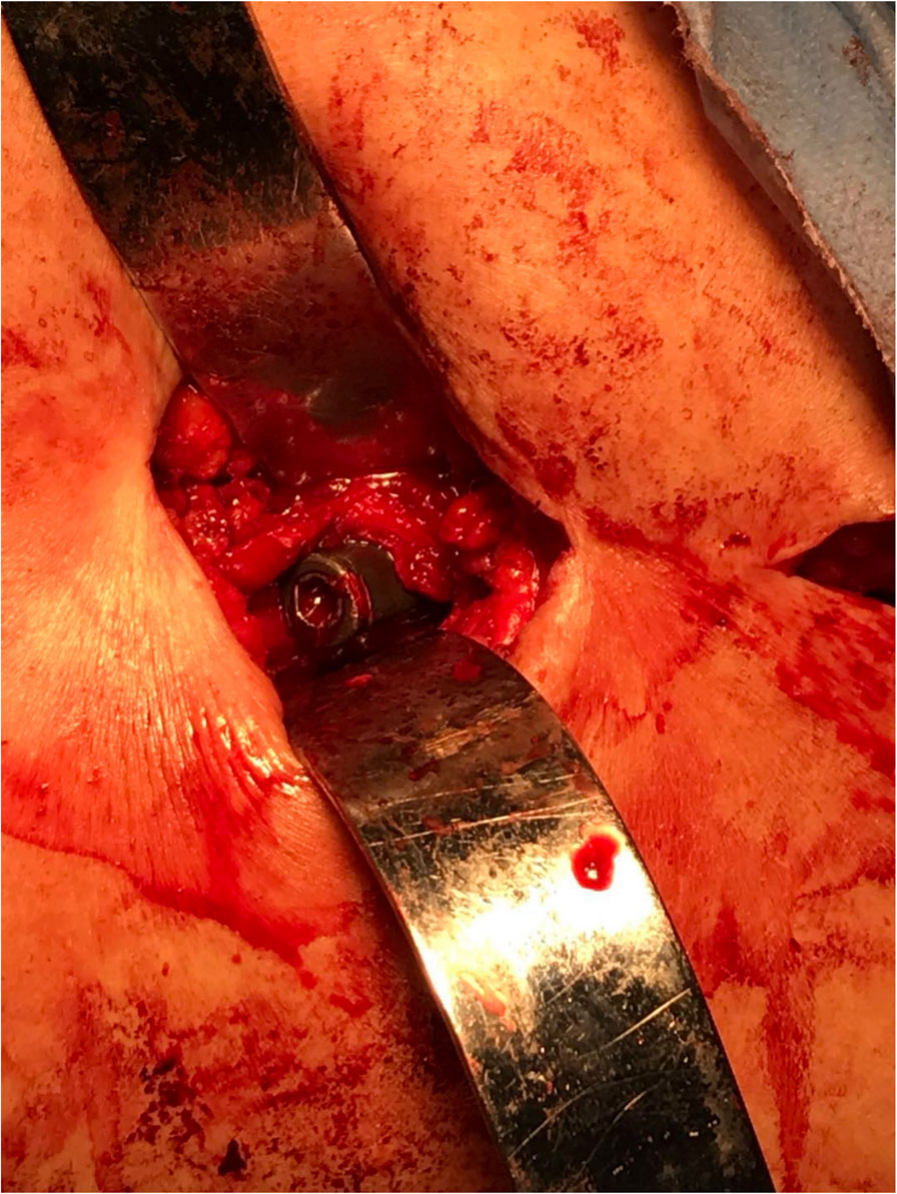
Intraoperative image

**Fig. 4:**
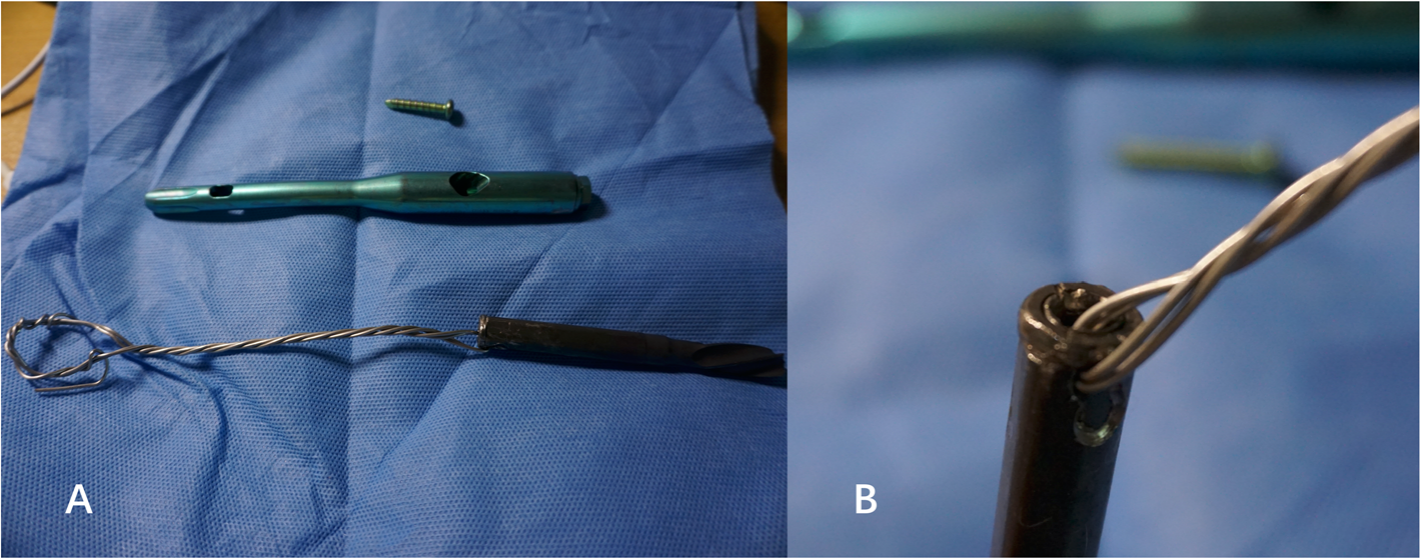
The steel wire loop was threaded through the helical blade (**a**), detailed structure of the helical blade end (**b**)

**Fig. 5:**
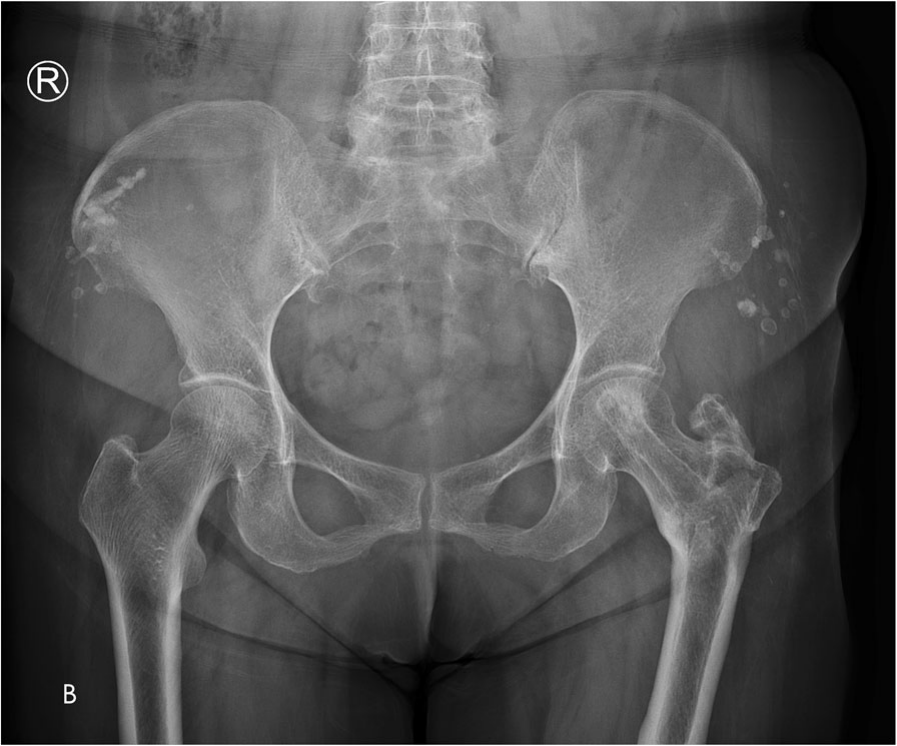
The anteroposterior radiograph of the pelvis after the PFNA removal

A 78-year-old lady sustained an intertrochanteric femoral fracture on the left side following a mechanical fall. She was treated with routine closed reduction, fixation with proximal femoral nail anti-rotation (PFNA). There were no postoperative complications, and the patient regained full range of motion of the left hip. Fifteen months later, the X-ray scan indicated the fracture had reached clinical healing (Fig. 1). Although the patient has been informed that the implant extraction is an elective procedure, she made a requirement to remove the implant. The procedure was performed under spinal anesthesia, utilizing the original lateral approach. During the first part of the procedure, the end cap and distal locking bolt were successfully removed. However, when we tried to remove the anti-rotation blade following the instructions (Fig. 2), it was found that the hexagonal socket slipped (Fig. 3) and could not lock up with the extraction screw for PFNA blade. Therefore, the locking blind nut could not dispatch with the main body of the anti-rotation blade. After loosening the attachment of the blade and the greater trochanter, locking pliers were used to pull out the blade. However, the pliers could not provide enough tensile. Not only that, multiple failed attempts were made to remove the nail with conventional techniques. Thus, a unicortical hole was drilled by a 5-mm tungsten carbide bur at the end of the blade, and a 2-mm double-strand steel wire was threaded through the previously drilled hole (Fig. 4 a and b). The wire was twisted and strongly tied up to the extraction screw by hard loop. Eventually, the blade was removed by applying blows of the combined hammer, in the direction of the blade. The post-operation X-ray (Fig. 5) showed the implants were utterly removed. The patient was encouraged to take full weight 2 weeks after surgery with anti-osteoporosis treatment.

## Discussion

The incidence of hip fractures in the elderly continues to increase drastically, and the proximal femoral nail anti-rotation is often used to treat intertrochanteric fractures. Taking into account of operation necessity and safety, PFNA removal is not a routine surgical procedure for the majority of patients, whereas complications like non-union and intractable regional pain of greater trochanter area are not extremely unusual, which are still removal indications [2, 3]. Compared with TFN, PFNA has helical blade end cap (most obvious distinction) according to the removal guide from Depuy Synthes [4]. The conventional blade removal process is well described in the manual. Firstly, 3.2-mm Guide Wire is inserted through the blade. Then, push the Extraction Screw for PFNA Blade over the guide wire and use gentle pressure to turn it counterclockwise into the PFNA blade. Once the surgeon sees “unlock” etching on the Extraction Screw, light hammer blows with the Detachable Slide Hammer are applied to remove the blade. It is worth noting that the helical blade is a split-lock design. Hence, the locking mechanism is vital to the bolt and removal process. Wang et al. described a case where helical blade could not be tightened and locked by the blade impactor as usual [5]. When the PFNA is taken out, the end cap of the main nail is relatively easy and reliable to be taken out by Hexagonal Screwdriver. However, the greater trochanter area, as well as the entry point of helical blade is a high-stress area, and the osteophyte proliferation is often severe. Then, osteotomes are used to expose the end of the blade. During this process, the external force may cause deformation of the blade end, which will result in unlocking failures in some cases. In this scenario, the firm-holding force provided by the blade often leads to removing difficulties. In the absence of special apparatus like expansion bolts or other destructive removal tools, we introduced a method which uses a tungsten carbide bur for drilling a single cortical hole at the end of blade. A double-strand steel wire is threaded through to bind the extractor and assorted slide hammer. This method can provide great holding power and can effectively remove the blade by applying gentle blow of the hammer along the anatomical direction of the femoral neck. This surgical procedure is very convenient to be used in patients with PFNA removal difficulties. We advocate the use of this elegant and simple pushout technique with the creation of a steel wire loop and use of a tungsten reamer in extraction difficult cases.

## Data Availability

Data sharing is not applicable to this article as no datasets were generated or analyzed during the current study.

## Abbreviations

PFNA: Proximal femoral nail anti-rotation
